# A government under an asbestos roof

**DOI:** 10.4103/0019-5278.40820

**Published:** 2008-04

**Authors:** Gopal Krishna

**Affiliations:** Ban Asbestos Network of India (BANI). E-mail: krishnagreen@gmail.com

Dear Sir,

This is to bring to your attention a debate has broken out in the pages of the International Journal of Occupational and Environmental Health (IJOEH) on the above subject. I am attaching the document for you. The original critique of the American College of Occupational and Environmental Medicine (ACOEM) was published in 2007 in this same journal.

Of interest to Indian readers is a letter on industry's influence on the Indian Association of Occupational Health (IAOH). He writes about his experiences as the scientific committee chair at a past IAOH conference. The website for the journal is www.ijoeh.com/index.php/.

Given the fact that Indian Journal of Occupational and Environmental Medicine is the official publication of IAOH, it would also be relevant to bring to your notice the way National Institute of Occupational Health (NIOH) has compromised its credentials by taking the fiscal support from the chrysotile asbestos industry to do a study that is to be used at the upcoming Rotterdam Convention meeting.

The Indian Government has traditionally preferred the interests of the asbestos-cement industry, consistently ignoring evidence of its harms. The report will enable the government to go to Rome in October 2008 and argue in favour of unrestricted trade of white asbestos under the Rotterdam Convention. India along with Canada and Russia is seen as the dirtiest player in the international negotiations to bring white asbestos in the prior informed consent (PIC) list of the convention, which will require exporting countries to obtain the prior consent of importing countries.

The study on which the ministry's report based is partly funded by the Asbestos Cement Product Manufacturers Association, which paid Rs. 16 lakh, whereas the government paid Rs. 43.66 lakh. What surprised occupational safety campaigners is the agency that conducted the study, the NIOH, which in the past has come out with studies defending workers’ health.

The study's terms of reference reveal the government's intent. Here is what the ministry's April 2006 letter demanded of NIOH: “The deliverables will include generation of data which would justify the safe standards of its usage and the reasons justifying its non-inclusion/or otherwise in the pic ambit.” The minutes of an April 2007 meeting of the ministry's review committee (half of which comprises asbestos industry representatives) gave NIOH a sharper focus: “It will specifically indicate as to how technology has made working conditions better. The same will include relevant photographs showing protective measures being undertaken.” The minutes of the Review Committee obtained recently through Right to Information Act dated 19 December, 2006 reads: “The report will be finalised after due discussions with the asbestos industry.” Another meeting minutes dated 18 April, 2007 reports that “…the results of the study which was underway could not be shared [with public] till the same was finalised.”

On April 18, 2007, the review committee felt the report did not adequately address the industry's requirements: “S Ganesan of ICC (Indian Chemical Council) and NIOH representatives will redraft/re-word the Kolkata report keeping in view the international sensitivities.” Ganesan's claim for the job: expertise in championing the pesticide industry in international negotiations. Clearly, a scientific study that is finalized after discussion with the corporate interests is grossly conflict of interest ridden and deserves to be scrapped. It is now time for Indian Journal of Occupational and Environmental Medicine to take note of it as well.

I would be glad to know your views on the goings-on.

*…any man's death diminishes me, because I am involved in mankind and therefore never send to know for whom the bell tolls; it tolls for thee.*
John Donne

